# Methods for stratification of person-time and events – a prerequisite for Poisson regression and SIR estimation

**DOI:** 10.1186/1742-5573-5-7

**Published:** 2008-11-14

**Authors:** Klaus Rostgaard

**Affiliations:** 1 Department of Epidemiology Research, Statens Serum Institut, Artillerivej 5, DK-2300S Copenhagen, Denmark

## Abstract

**Introduction:**

Many epidemiological methods for analysing follow-up studies require the calculation of rates based on accumulating person-time and events, stratified by various factors. Managing this stratification and accumulation is often the most difficult aspect of this type of analysis.

**Tutorial:**

We provide a tutorial on accumulating person-time and events, stratified by various factors i.e. creating event-time tables. We show how to efficiently generate event-time tables for many different outcomes simultaneously. We also provide a new vocabulary to characterise and differentiate time-varying factors. The tutorial is focused on using a SAS macro to perform most of the common tasks in the creation of event-time tables. All the most common types of time-varying covariates can be generated and categorised by the macro. It can also provide output suitable for other types of survival analysis (e.g. Cox regression). The aim of our methodology is to support the creation of bug-free, readable, efficient, capable and easily modified programs for making event-time tables. We briefly compare analyses based on event-time tables with Cox regression and nested case-control studies for the analysis of follow-up data.

**Conclusion:**

Anyone working with time-varying covariates, particularly from large detailed person-time data sets, would gain from having these methods in their programming toolkit.

## Introduction

Some of the most common analytic epidemiological methods are based on calculating and comparing rates of occurrence of events in follow-up studies. Some of these methods work in exact, continuous time (e.g. standard Cox regression), while others are based on the assumption of constant rates of occurrence of events within strata of the data, so that tables of person-time at risk and number of events stratified by various factors, known as event-time tables, are sufficient for such analyses. Standardised mortality ratios (SMRs), standardised incidence ratios (SIRs), Poisson regression and associated additive rate models all require such data sets [1-3]. Such methods may be increasingly important in the analysis of follow-up studies [4].

The stratification of individual follow-up time by age, calendar period and other variables, and subsequent aggregation of follow-up time and events over individuals within these strata, does not pose much of a challenge theoretically. Nevertheless, "the creation of an adequate event-time table is often the most difficult aspect of carrying out analyses of rates using Poisson regression" [1]. To begin with, there is often a non-trivial amount of bookkeeping involved, along with the need for handling of anomalies in the raw data that arise due to the discretisation of time, typically with days as the finest unit of time. Therefore, there is a desire for a methodology and a user interface that permit fast, bug-free programming, as well as for easily readable programs and documentation. Furthermore, the computing resources required for creation of detailed event-time tables from large data sets may be prohibitive. This problem is even more likely to occur in the common situation where there are several different outcomes of interest, with accompanying variations in person-time.

We will present a new method for handling this situation, taking advantage of the fact that typically most follow-up time will be free of all outcomes. We will also discuss our preferred solutions to many of the typical problems encountered in event-time table creation and provide a new vocabulary to characterise and differentiate time-varying factors. The tutorial uses a macro for the most standardised tasks in the creation of event-time tables. Through its data interface and user interface, the macro offers new and improved programming possibilities for handling situations with many outcomes simultaneously or with time factors representing current status or with follow-up when the origin-defining event has not yet occurred or when using time factors growing alternately at the speed of time or not at all (e.g. cumulative employment). The macro and illustrative examples are coded in SAS [5]. Since in many applications of Cox regression, we need to stratify individual follow-up time by factors like number of children and place of residence, we will also show the usefulness of the macro and the methodology for producing data for Cox regression analyses of this type.

We think the presented methodology supports the creation of bug-free, readable, efficient, capable and easily modified programs for making event-time tables. There are many other macros, tools and programs available for making event-time tables. However, most of these are specialised or limited in their capabilities, while others, such as epicure [6] and OCMAP [7], require additional costly software to run. In our view, the methods described in this paper and the attached freely available macros [8], form the best available starting point for analysis of follow-up studies based on event-time tables in a popular general-purpose statistical software package.

Few published papers [9,10] contain general event-time table methodology, in the sense of proposing ways of dealing with all types of time-varying factors. Most papers in the field have presented software with limited or specialised capabilities. Therefore, in our experience, many users of these techniques have often had little guidance in event-time table construction beyond help from experienced colleagues. This paper focuses on examples of the most common approaches to creation and analysis of event-time tables. Executable source code for the examples is included in [8].

## Tutorial on creation of event-time tables

**Figure 1:**
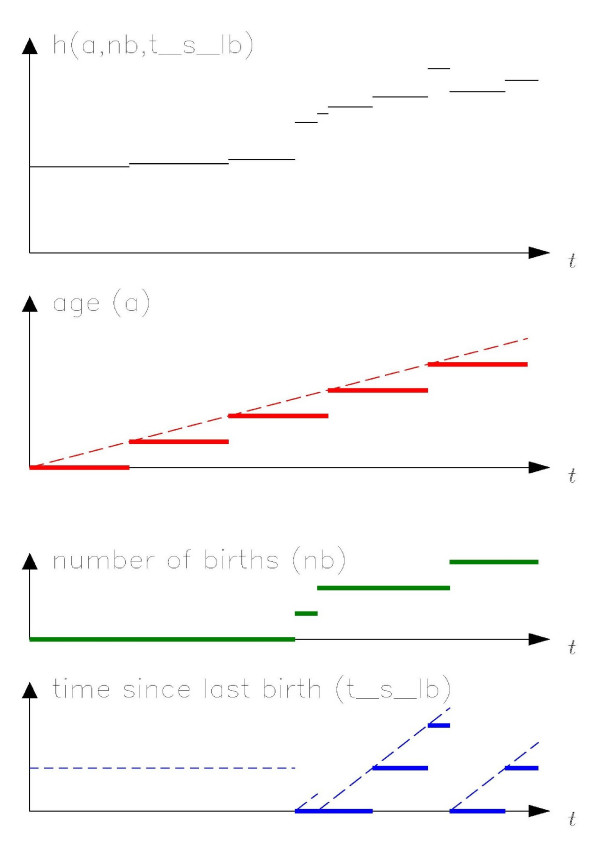
**A model for which parameter estimation can be based on an event-time table**. We assume that the incidence rate *h(...) *for some event depends only on three time-varying factors, namely age and time since last pregnancy, suitably categorised, and number of births. Each combination of levels of the three time-varying factors defines a stratum within which the incidence rate is constant, leading to piecewise constant incidence rates. The graphs show the changing values of the time-varying factors ("exposures"), the changing values of the stratified time-varying factors (the fat lines) and the resulting incidence rate during follow-up of one person.

Figure 1 shows a model based on event-time tables. The common theme to these models is the assumption that the hazard rate of some event for any individual varies over time according to factors that are either discrete (e.g. number of births) or discretised versions of underlying continuous variables (e.g. age). In order to draw statistical inferences, we do not need individual records for each cohort member, but can instead use tables of person-time at risk and number of events stratified by time-dependent factors; such tables are known as event-time tables [1]. An event-time table, then, is merely a data set used as input for a statistical analysis, and conveys useful information in tabular form only in extremely simple situations.

The process of converting raw data to results using event-time tables consists of four steps:

*1) pre-processing *(retrieval and formatting of data as input to step 2),

*2) stratification *by time-dependent factors (including constants) *and aggregation*,

*3) post-processing *(e.g. generating data for SIR analysis from age- and sex-specific rates and risk time and events stratified accordingly) and

*4) analysis *(e.g. estimation and testing).

Sometimes, some of these steps may be trivial. This text presents a macro (which we refer to as "the macro") for performing some or all of step 2. We provide only limited discussion of step 4, as it involves statistical considerations that may differ across studies. The following examples show what happens to the data records from one or two persons to illustrate the workings of the macro in conjunction with other necessary programming steps. These examples do not illustrate real world applications and the reader will not see the effect of aggregation.

### Single outcome follow-up study: Pre-processing

**Figure 2:**
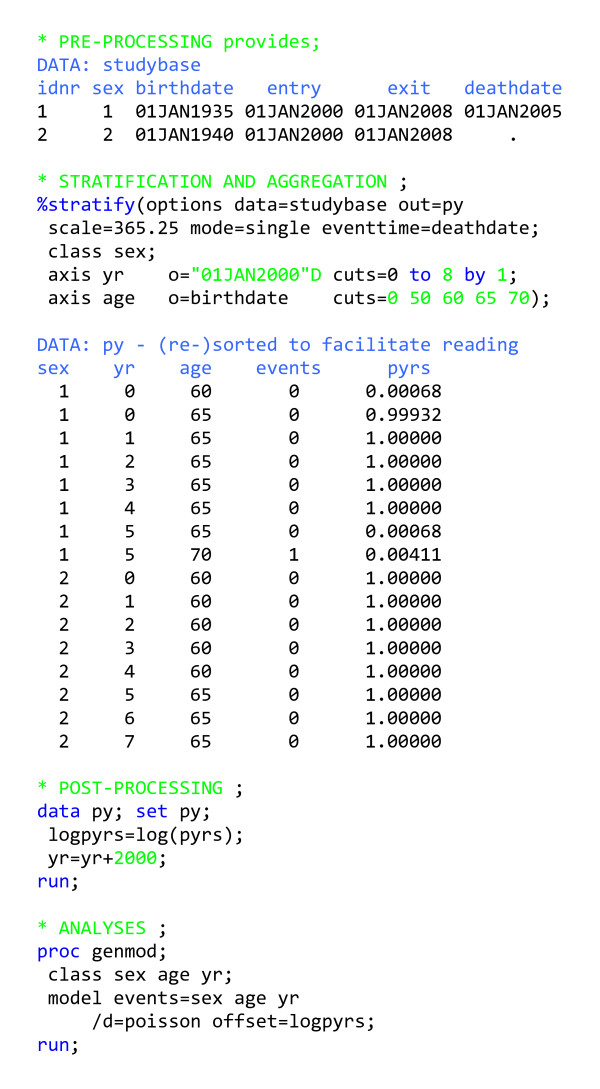
Program outline for analysing follow-up data with one outcome using Poisson regression.

In this example (figure 2), we follow a cohort of persons from some time point to a particular outcome (here, death), with the data to be analysed using Poisson regression. We assume that each record (observation) in the input data set contains all relevant information about a person being followed. We want each person to enter the study at a time point captured by the variable *entry *and exit from the study at a time point captured by the variable *exit*. In our example, *entry *is the start of the study or birth, whichever occurs later, and *exit *is the point at which follow-up ends for a reason other than the occurrence of the outcome (e.g. end of study, migration, etc.). The macro then removes persons from risk if an outcome occurs before *exit*. The interval *entry *to *exit *is interpreted as including *entry *and excluding *exit*. We assume that if an outcome occurs, it occurs at a time captured by the variable *deathdate*; otherwise this variable is set to missing.

### Single outcome follow-up study: Stratification and aggregation

The user interface for the macro (%stratify) is designed to resemble that of a SAS procedure and is therefore not typical of a SAS macro. However, all macro statements and some of the statement components become macro variables within the macro. In the notation, we have italicised SAS variables, data sets and values, and capitalised macro variables. The macro takes the input data set (*studybase*) and stratifies the time intervals from *entry *to *exit *and the event times by year and age. A stratified version of time since some event is obtained by inventing a time axis with the event as the origin and some cut points along the time axis, where we denote the interval from one cut point to the next by the value of the interval's left endpoint. Each time axis is given a variable name (here, *yr *and *age*), an origin (e.g., *birthdate *for the age axis) and some cut points, relative to the origin. The input data time points (*entry*, *exit*, the various origins) for a given person have to be on the same time scale, with calendar time as the obvious choice. By using the SCALE macro variable, it is possible to let the input data time points be SAS dates, while the specification of the cut points is in time units such as years (obtained by setting SCALE = 365.25, the default value). Thus in this example, where the input data time points are SAS dates, the second time axis is stratified into 0–49, 50–59, 60–64, 65–69, 70+ years since birth, with the variable *age *receiving the value of the interval's left endpoint. Note that the right-most interval for each time axis is always open-ended on the right.

We end follow-up on *exit *or the date of occurrence of the outcome (+ 1), whichever occurs first. Adding a day to the date of occurrence of the outcome ensures that all persons who experience an event contribute at least one day of risk time. The size of this overhang is specified by the macro variable GRANULARITY, which by default is set to 1. In most situations, we do not know when things happen with greater precision than a day, so it is reasonable to assume that people are at risk throughout the day on which the outcome occurs. We signal our intent to end follow-up upon occurrence of a single outcome by specifying MODE = single. We also need to specify which variable captures the time of occurrence of the outcome; this is done by specifying the value of the macro variable EVENTTIME.

Finally, the output from the macro is aggregated, meaning that the output data set (which is the event-time table, here called *py*) is the smallest possible summary of person-time and events stratified by the specified factors. The macro stores the number of events and the person-time in variables called *events *and *pyrs*, respectively. Thus in this example, *py *contains one record for each combination of the variables *sex*, *age *and *yr *with either *events *> 0 or *pyrs *> 0. The macro obeys the SAS convention that if no input data set is specified, the last data set created will be used as input. Similarly, if no output data set is specified, the macro will overwrite the last data set created. In this example, the small deviations from one year at risk observed in each output record are due to the incongruence of actual time and calendar time, and the granularity.

### Single outcome follow-up study: Post-processing

This step is trivial in this example, as we only construct a variable to be used in the analysis and recode another variable so it has a recognizable appearance.

### Single outcome follow-up study: Analysis

Suppose we want to estimate incidence rate ratios for the effects of age adjusted for sex and calendar year. In terms of a parametric function for the rates, the log likelihood under the assumption of piecewise constant hazard rates is equivalent to the log likelihood that would arise if the event counts in the table were independent Poisson random variables. Thus, Poisson regression can be used to estimate the parameters in this model [1,3].

The model formulated in figure 2 specifies that the expected number of events for the i^th ^observation in *py *is exp(X_i_β)T_i_, with independence between observations and variation equal to the expected number of events, with X_i _specified in the model statement and T_i _denoting the risk time, specified by the offset. Incidence rate ratios and confidence limits are immediately available by exponentiating the parameter estimates and their confidence limits. Additional modelling, stratifying and testing is also possible.

### Standardised incidence ratios for multiple outcomes: Pre-processing

**Figure 3:**
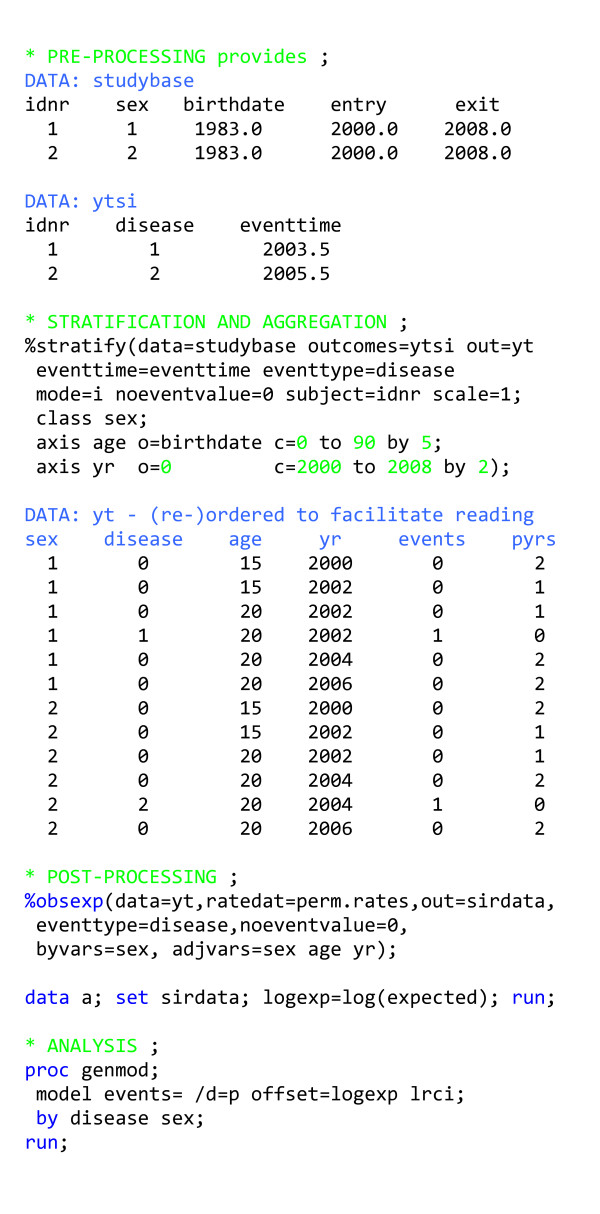
Program outline for analysing follow-up data using standardised incidence ratios for many different outcomes simultaneously.

In this example, we will calculate SIRs for many different disease outcomes for the same cohort (figure 3). We suppose each record in the data set (*studybase*) contains all relevant information about a person in the cohort except the outcomes, which are in a different data set (*ytsi*) where each observation contains the first occurrence of a given disease (*disease*), the time of occurrence of the disease (*eventtime*) and a unique person identifier (*idnr*). To simplify the presentation in this and the following examples, all input data time points will be in decimal calendar years, thereby avoiding the consequences of the incongruence of actual time and calendar time.

In the construction of the event-time table, we should mimic the process by which events and time at risk for the reference rates have been created. Reference rates are commonly calculated using incident cases and person-time based on population figures, ignoring prevalent cases. In that case, follow-up should not end with the occurrence of an outcome.

### Standardised incidence ratios for multiple outcomes: Stratification and aggregation

We assume that sex-, age, and period-specific incidence rates for each disease in a given population are available, so we stratify in the same way that the available reference rate data set is stratified (*perm.rates*). Within the macro, data (*studybase*) and outcomes (*ytsi*) are linked using the personal identifier (specified by SUBJECT = *idnr*). We also need to specify EVENTTIME, the variable capturing the time of occurrence of the outcomes. Records where *entry *≥ *exit *are ignored by the macro. Similarly, the macro ignores events for which there is no matching segment of time at risk, requiring that the data set (DATA = ...) contain a record with *entry *≤ EVENTTIME <* exit *for the same person (SUBJECT = ...) in order for the event to be tallied in the output data set (OUT = ...). This output data set contains two types of records: those containing only risk-time (*events *= 0) and those containing only number of events for each disease (*pyrs *= 0). To distinguish between the two types of records in a simple way, we assign a special value to the disease variable for risk-time contributions without a disease event (NOEVENTVALUE = 0 in this example). We specify that we intend to produce this type of output by setting MODE = i; for this mode of operation, we also have to specify the variable distinguishing the multiple outcome events we want to study (EVENTTYPE = *disease*).

### Standardised incidence ratios for multiple outcomes: Post-processing

We make use of another macro (%obsexp) to calculate the observed and expected number of events from an event-time table in this format. This macro produces an output data set (*sirdata*) containing the observed and expected number of events and person-time at risk for each combination of the levels of the variables specified in the EVENTTYPE and BYVARS macro variables (*disease*, *sex*). In order for the macro to calculate the expected number of events, it needs a data set containing rates (*perm.rates*) stratified by the variables specified in EVENTTYPE and ADJVARS. Obtaining correct results requires variable names and coding to be the same in the input data for this macro and the rate data set. Extra programming may be required to standardise the data sets in this way.

### Standardised incidence ratios for multiple outcomes: Analysis

The output from proc genmod provides the log(SIR) and accompanying likelihood ratio-based confidence limits for each combination of disease and sex. Using generalised linear modelling makes it possible to obtain likelihood ratio-based confidence limits, which are preferable even to accurate approximations such as Byar's formula [ref [3], page 68–71], which can no longer be justified by computational expediency. A modelling approach is also preferable when we want to test the homogeneity of the SIRs over categories.

### Competing risks follow-up study

**Figure 4:**
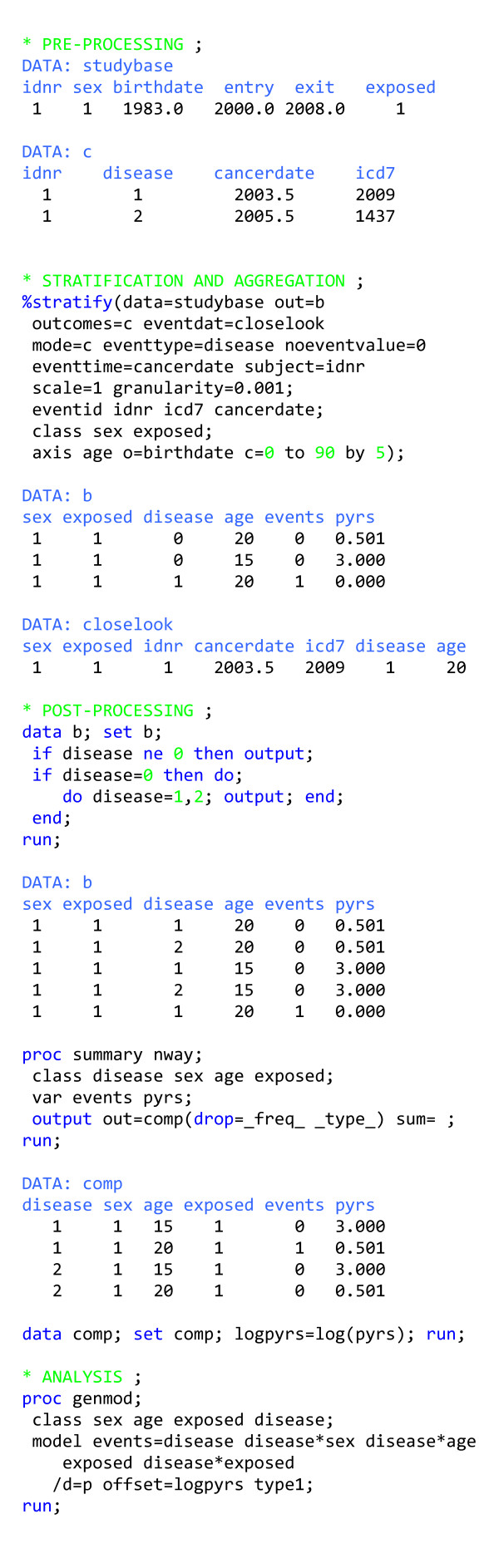
**Program outline for analysing competing risks**. The competing risks analysis presented here is a general one and is not restricted to the situation where the outcomes preclude additional events from occurring. Instead, we analyse competing risks for the occurrence of the first outcome event.

This example shows how to analyse competing risks (figure 4). Here the follow-up ends upon censoring or the first occurrence of an event for a given person i.e. the follow-up time is the same for all events considered. We signal our intention to do this type of analysis by setting MODE = c, and need to introduce a factor with categories denoting possible outcomes (EVENTTYPE = *disease*), with a special category to denote follow-up time (NOEVENTVALUE = 0). After stratification and aggregation, we can separate the output data set (*b*) into a person-time data set and an event data set, based on the value of the above-mentioned factor (*disease*). We make a copy of the person-time data for each outcome and combine these data with the event data generated for that outcome to generate sufficient data for this type of analysis [11]. In order to test whether the exposure acts differently on the various diseases considered as outcomes, a disease-exposure interaction term is added to the model statement in proc genmod. For example, the various diseases could be subtypes of a single cancer.

In this example we have also used EVENTDAT = *closelook *and EVENTID *idnr icd7 cancerdate*, which together cause the macro to produce an extra data set (*closelook*) that contains a record for each outcome event with all the variables produced for the event-time table, except *pyrs *and *events*, plus whatever extra variables are specified by EVENTID. This feature is useful for checking exactly which events contribute to which strata. Alternatively, we may be performing an SIR analysis based on broad disease categories, because those are the background rates we have, but in actuality, our hypothesis is that the exposure of interest will induce a surplus of a specific disease subtype, and we can then have an informal look using the EVENTDAT option.

### Multiple outcomes follow-up study

**Figure 5:**
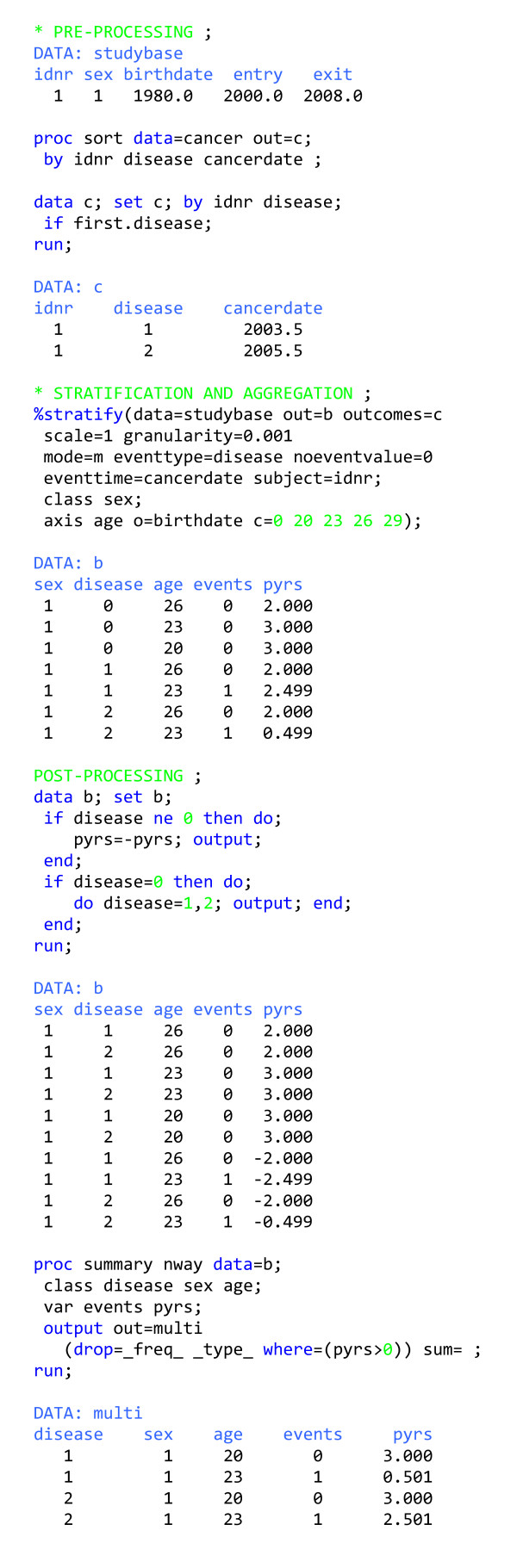
Program outline for generating a data set for Poisson regression for many outcomes simultaneously.

Finally, suppose that we wish to follow several outcomes where follow-up for a given outcome ends when the outcome occurs (figure 5). For typical outcomes, most follow-up time will be disease-free, so the method that is computationally simplest for generating the event-time table is to calculate all the potentially available person-time once and then for each disease subtract the person-time contributions after the occurrence of the event (i.e., after EVENTTIME + GRANULARITY). Part of this calculation is done within the macro (specified by MODE = m), and part of it has to take place in the post-processing step where the value (≠ NOEVENTVALUE) of the variable *disease *(EVENTTYPE) signals the excess disease-specific risk time.

The usual way to generate event-time tables for multiple outcomes is to generate output data sets for one outcome at a time (possibly via code packaged into a macro) and then combine all these data sets for analysis. This usually takes much longer than our alternative and saves little, if any, coding work.

### Why aggregate the event-time table?

We compared our method (aggregating the output from the stratification step) with a no-aggregation method on a typical PC using data from Nielsen et al. [12]. In this study, a cohort of Paul-Bunnell-tested persons was followed for multiple sclerosis, the effect of positive versus negative Paul-Bunnell status was estimated, adjusting for calendar period, sex, attained age and time since test. The standard output from %stratify contains 11,113 observations. It takes 2 seconds to run %stratify and 1 second for proc genmod to produce parameter estimates. If we add personID to the list of class variables (corresponding to no aggregation), the event-time table produced by %stratify now contains 1,740,876 records, and it takes proc genmod more than 2 minutes to produce parameter estimates.

In the appendix, we compare different aggregation approaches and conclude that the macro's new default method (METHOD = fst) is fastest and that the typical analyst is unlikely to need any of the other aggregation methods available in the macro. However, if the default method requires too much RAM, METHOD = sum or METHOD = chunk could be used instead (see appendix for further details).

### Types of time-varying factors

We have found only two papers [9,10] that cover exact stratification by all types of time-varying factors. The method proposed by Wood *et al*. [9] is simple, robust and general, covering all types of time-varying factors in the same way. Our main criticism of this approach is that as the number of persons in the study and the complexity of the exposure history to be represented increase, the time and temporary memory required become prohibitive; a computationally more efficient approach is therefore in order. Macaluso [10] presents several methods, one of which is general enough to handle all types of time-varying factors. This approach, however, has the same drawbacks as the approach by Wood *et al*. [9], although not to the same extent.

We have invented a new classification of time-varying factors, extending and modifying the classification used by Macaluso [10]. We consider the new classification to be helpful in organising and programming the event-time table generation, because there is a clear correspondence between types of time-varying time factors and the tools and programming techniques that can be applied to stratify them.

We distinguish between four types of time-varying factors (zero-rate, unity-rate, bit-rate, and flex-rate time factors), defined by the possible rates of change in each. Zero-rate time factors include factors which are constant (e.g. sex, race) and factors which are constant between status changes (e.g. number of pregnancies to date, number of jobs held to date, current marital status). Unity-rate time factors, such as age, period, and time since last pregnancy, change at a dimensionless rate of 1, because the changes in the numerator and denominator are the same. In bit-rate time factors, the rate of change will be 0 in some follow-up intervals and 1 in others. A typical example is cumulative length of employment, growing at a rate of 1 during periods of employment and a rate of 0 during periods of unemployment. With flex-rate time factors, the rate of change can take any value. A typical example is the cumulative number of cigarettes smoked to date. This nomenclature places fewer time factors in the most complicated category (here, flex-rate) than does Macaluso [10] and is more descriptive. Using the example in figure 1, the hazard function depends on a, nb, and t_s_lb, which are stratified versions of the time factors age, number of births and time since last birth, respectively. Age and time since last birth (when defined) change at the same rate as *t *(i.e., with slope = 1) and therefore are unity-rate time factors, whereas number of births is constant between births and is therefore a zero-rate time factor.

Zero-rate time factors that are not constant should be handled by stratifying the follow-up time into intervals where these variables are constant, prior to stratification by other types of time-varying factors. Note that zero-rate time factors are distinct from other time-varying factors in that for zero-rate time factors, the input data contain time points at which the value of the stratified version of the time factor *might *change, whereas for other time-varying factors, we must calculate the time points at which the value of the stratified version of the time factor *does *change.

The only complication with unity-rate time factors occurs if the origin of a time scale is either absent or changes during follow-up, as it may for unity-rate time factors such as time since last pregnancy or time since last hire. In such instances, the input data must be stratified into intervals with only one (possibly missing) origin for each time scale before stratification by unity-rate time factors; in other words, the input data should first be stratified according to the zero-rate time factor "last occurrence of the origin-defining event". When a time scale origin is missing, the macro assigns the value of the largest specified cut point for that time axis to the relevant variable for the given event or risk-time contribution. For example, suppose that we are following a cohort of women for incident ovarian cancer and want to include time since last pregnancy as an explanatory variable. Specifying e.g. 0, 1, 2, 3, 5, 7, 10, 15, 25, and 999 years as cut points for the time since pregnancy time axis ensures that only contributions from never-pregnant women are assigned to the 999 category, which also forms a natural reference category when analysing this factor as a categorical variable.

In principle the procedure for dealing with unity-rate time factors is easily extended to allow for handling of bit-rate time factors. We divide the follow-up time into intervals with constant rates of change in the bit-rate time factors and process these intervals in chronological order, carrying over pertinent information from previous intervals to the one being processed.

With flex-rate time factors, stratification of follow-up time requires situation-specific programming. To minimise programming time and for ease of documentation, we strongly recommend doing this as part of the pre-processing step, with the output identical to that generated for a zero-rate time factor. As a general methodology, Wood et al. [9] suggest stepping through the follow-up time for each individual in steps corresponding to the finest granularity of time in the data (typically days), in each step identifying one stratum to assign the risk-time contribution to and outputting the results. While simple, and therefore robust, such an approach can rapidly generate enormous amounts of temporary data. However, this approach may be useful for identifying time points where the value of the stratified version of a flex-rate time factor changes and outputting these and relevant attributes to be combined back into the raw data in the same way as other such points of change. In our experience flex-rate time factors are rarely used and require ad-hoc programming, so we will not discuss them any further.

### Handling zero-rate and bit-rate time factors with the macro

In our experience, almost all non-constant zero-rate time factors requiring stratification can be categorised into a few distinct categories (has the exposure occurred?, age at last exposure, age at first exposure, number of exposures to date, etc.). The macro takes advantage of this feature to stratify zero-rate time factors in a simple way. The user provides one or more exposure data sets (EXPDATS ...), each of which contains a variable containing an individual person identifier (SUBJECT = ...) allowing for linkage to the studybase; a character variable signalling which exposure event has occurred (EXPEVENT = ...); a variable indicating the time when the exposure event occurred (TIME = ...); and a variable containing a numeric (VALUE = ...) or character (VALUEC = ...) attribute of the exposure event, plus a line of code generating the stratified zero-rate time factor (ZRTF ...). The alternative to all this is repetition of very similar pieces of code for each factor outside the macro. Furthermore combining time-varying exposures from different data sets is traditionally non-trivial.

**Figure 6:**
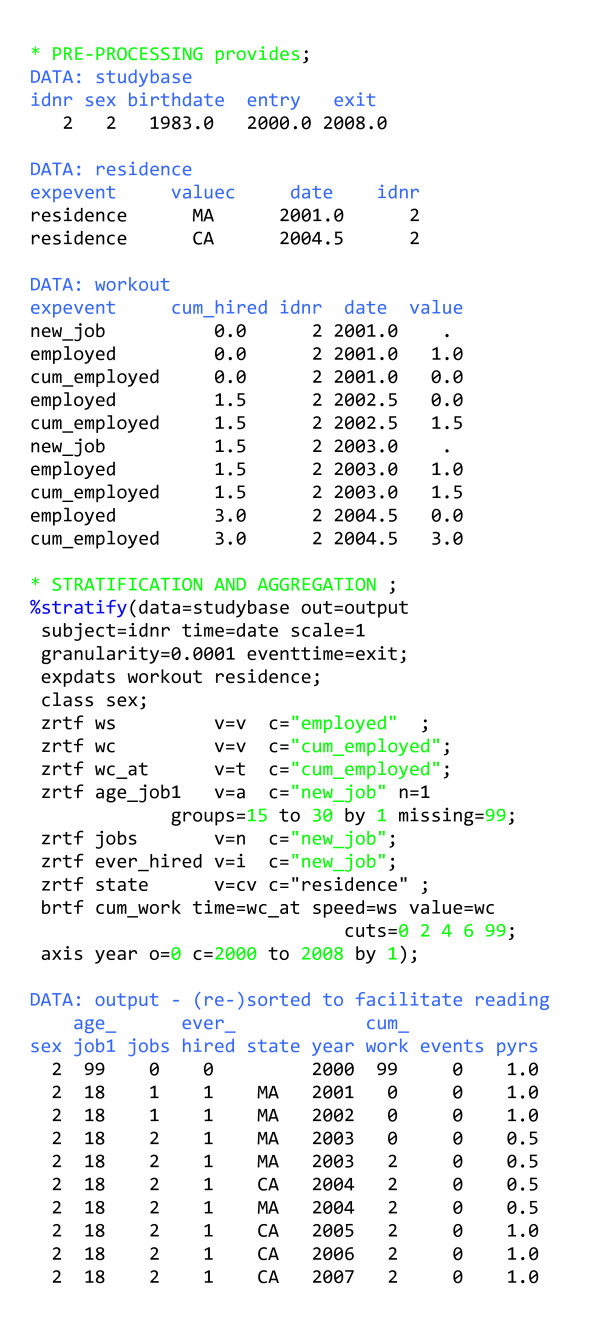
Stratifying follow-up time by zero-rate time factors, bit-rate time factors and unity-rate time factors.

In figure 6, we follow one person (in *studybase*) and have two data sets containing exposures (EXPDATS *workout residence*). Since we use the default values for EXPEVENT, VALUE and VALUEC, they need not be specified in %stratify. The line ZRTF *everhired *... generates a variable (*ever_hired*) that is an indicator (v = i) for the exposure event (c = "new_job"); for each person, *ever_hired *is initialised to 0, and the first time the content of EXPEVENT is "new_job", *ever_hired *becomes 1. In the line ZRTF *state *... we generate a character variable which takes the value of VALUEC (v = cv) whenever the exposure event is "residence". This variable is initialised to be blank and then retains the value corresponding to the most recent occurrence of the exposure event "residence" for that person. If we had added n = 1 to this line, the variable would have retained the value corresponding to the first occurrence of "residence"; if we had added n = 2, it would have retained the value corresponding to the second occurrence of "residence", and so on. The default is n = L (for last).

In the first five ZRTF statements in figure 6, we see some other values that the variables can take: the value (v = v), the time (v = t) and the age (v = a) at which the exposure event occurred. In the fourth ZRTF statement, n = 1 yields the age at the first job, with age categorised in 1-year intervals from 15 to 30 years of age. The grouping in a ZRTF statement works somewhat differently from that in the AXIS statement. All values are rounded down to the nearest cut point, and all values below the smallest cut point (along with missing values) are set to missing. For the age calculation to work, we need the date of birth, given by BIRTHDATE = birthdate, which is the default and so not explicitly specified.

Some of the variables generated by the ZRTF statements do not appear in the output. The macro has built-in logic that decides whether such variables were needed only as intermediate variables (e.g. the origin of some time scale) or are of primary interest and therefore should be included in the event-time table. All variables used to construct bit-rate time factors in BRTF statements and all variables generated with ZRTF statements with value = t are dropped from the output unless saved by inclusion in the CLASS statement. (More about bit-rate time factors appears below.)

The macro can do many things, at the cost of having many macro variables to assign values to. Table 1 serves as a guide for what must be specified when in the first statement, and table 2 gives an overview of the syntax for the remaining statements. For further details, see [8]. In order to reduce the slope of the learning curve and improve productivity [13], we have tried as much as possible to give the macro the appearance of a typical SAS procedure, with entries that look like procedure options (all macro variables with a single value (table 1)) and entries that look like procedure statements with several arguments (table 2), and we have organised our examples accordingly.

**Table 1:** Simple macro variables in the stratify macro i.e. options.

macro variable	default value	needed when	meaning
DATA	&syslast	mandatory	input data
OUT	&data	mandatory	output data
OUTCOMES		MODE = m	outcome events input data
EVENTDAT			outcome events output data
EVENTTIME	eventtime	mandatory	time of outcome event
SUBJECT		OUTCOMES or EXPDATS used	person ID for record linkage
TIME	time	zrtf-stmt	time of exposure events
EXPEVENT	expevent	zrtf-stmt	exposure event
VALUE	value	zrtf-stmt v = v	exposure event attribute, numeric
VALUEC	valuec	zrtf-stmt v = cv	exposure event attribute, character
BIRTHDATE	birthdate	zrtf-stmt v = a	birth date
SCALE	365.25	mandatory	scale relation between time units for time points and cut points/PYRS
GRANULARITY	1	MODE = s,c,m	follow-up ends at EVENTTIME + GRANULARITY
EVENTTYPE	eventtype	MODE = c,i,m	type of outcome
NOEVENTVALUE		MODE = c,i,m	value of eventtype for person-time
MODE	s(ing(le))	mandatory other values = c(omp(eting)) i(ntens(ity)) m(ult(iple))	when and how follow-up ends
METHOD	fst	mandatory other values = arr sum chunk noagg	method of aggregation or no aggregation (noagg)
CHUNKSIZE	5000	METHOD = chunk	see [8]

**Table 2:** "Statements" and their components in the stratify macro.

**statement **or statement component	Comment
**ZRTF **varname	varname must be a valid SAS variable name.
l(en(gth)) =	the length of varname. By default, 8 for numeric variables and the maximum length of VALUEC in EXPDATS for character variables.
c(ond(ition)) =	the value of the character variable EXPEVENT that will trigger the (re-)evaluation of varname. There are restrictions on the value of this variable to avoid it interfering with the macro, but a valid SAS variable name will always work. It must be enclosed in "".
n =	the occurrence of the condition that will trigger the (re-)evaluation of varname. Possible values are f, first, l, last, or any integer > 0, where 1 means first, 2 second etc. Last is the default.
v(al(ue)) =	the value of varname before grouping. Possible values are: v (value at occurrence of condition = and n=) s (sum of values at occurrence of condition=) i (indicator that condition = and n = has occurred) n (number of occurrences of condition=) cv (valuec at occurrence of condition = and n=) t (time at occurrence of condition = and n=) a (age at occurrence of condition = and n=).
g(roups) =	for a numeric variable, the specification is X to Y by Z or X Y Z W .... or a format. For a character variable, it is a format. No grouping occurs unless specified.
m(iss(ing))=	value of varname when missing. If varname is a character variable, it must be enclosed in "" and the same restrictions as for condition apply.
**BRTF **varname	varname must be a valid SAS variable name.
t(ime) =	variable containing the time point at which speed and value are evaluated.
s(peed) =	variable containing the rate of change (0 or 1) of the underlying bit-rate time factor (from time and onwards).
v(al(ue)) =	variable containing the (ungrouped) value of varname at time.
c(uts) =	X to Y by Z or X Y Z W ....
**AXIS **varname	varname must be a valid SAS variable name.
o(ri(gin)) =	variable or constant containing the origin of the time scale
c(uts) =	X to Y by Z or X Y Z W ....
s(peed) =	variable or constant containing the rate of change. The rate of change is always 1 unless this variable or constant is set to 0.
**EXPDATS**	exposure data sets.
**EVENTID**	extra variables to include in EVENTDAT.
**CLASS**	stratifying variables to appear in OUT retained from DATA.

Internally, the macro works as follows: It 1) gathers all relevant exposure variables from the relevant data sets, links them and sorts them; 2) stratifies the data according to zero-rate time factors; 3) links outcome events with the stratified data to characterise the outcomes; 4) combines and manipulates the exposure and outcome data according to the value of MODE, and carries out the final preparation for handling bit-rate time factors; and 5) stratifies the data according to unity-rate and bit-rate time factors and aggregates the results. So ZRTF variables (*ws*, *wc*, *wc_at*, *age_job1*, *jobs*, *ever_hired *in figure 6) are always generated before BRTF variables (*cum_work*), which are always generated before AXIS variables (*year*), no matter how they are ordered in %stratify. We have tried to make the macro as clever as we could to facilitate use. For example, when MODE = s or c, the outcome data can reside in DATA or OUTCOMES, but if the latter is specified, all outcome data will be taken from there. When VALUEC has different lengths in different source data sets, it will be retrieved in a variable with the length of the longest VALUEC. Furthermore, the macro only retrieves required variables.

Bit-rate time factors are generally more cumbersome to handle than unity-rate and zero-rate time factors. In the BRTF statement (figure 6, table 2), the user must specify the value of the bit-rate time factor (value = *wc*) whenever there is a change in the value (0 or 1) of the variable describing the rate of change (speed = *ws*), along with the time at which this change occurs (time = *wc_at*). By providing this information in ZRTF variables, we ensure that the data are stratified into intervals in which the rate of change in the bit-rate time factor is constant. The macro calculates the value of the resulting zero-rate time factor at entry into the intervals and specifies an origin defined as time at entry minus this value. It then generates a variable named from (BRTF ..., *cum_work *in figure 6) that is treated as either a unity-rate time factor or a constant, depending on the rate of change, and stratified according to (cuts = ...). Since this process can be cumbersome, the generation of relevant input data set records in particular (in *workout*, in figure 6), the macro also offers shortcuts. If value = ... in the BRTF statement is not specified, the macro assumes the value variable is zero up to and including the time point when the speed variable is first encountered. From then on, the macro calculates the values of the value variable based on the alternating values of the speed variable (speed = ...). When value = ... is not specified in the BRTF statement, it is also unnecessary to specify time = .... The simplifying assumption that the value of the value variable is zero before the speed variable is set should be applicable in most cases, in which case we only need to generate the speed variable in a ZRTF statement and use it in a statement such as: BRTF varname speed = speedvar cuts = ....

### Output for Cox regression

**Figure 7:**
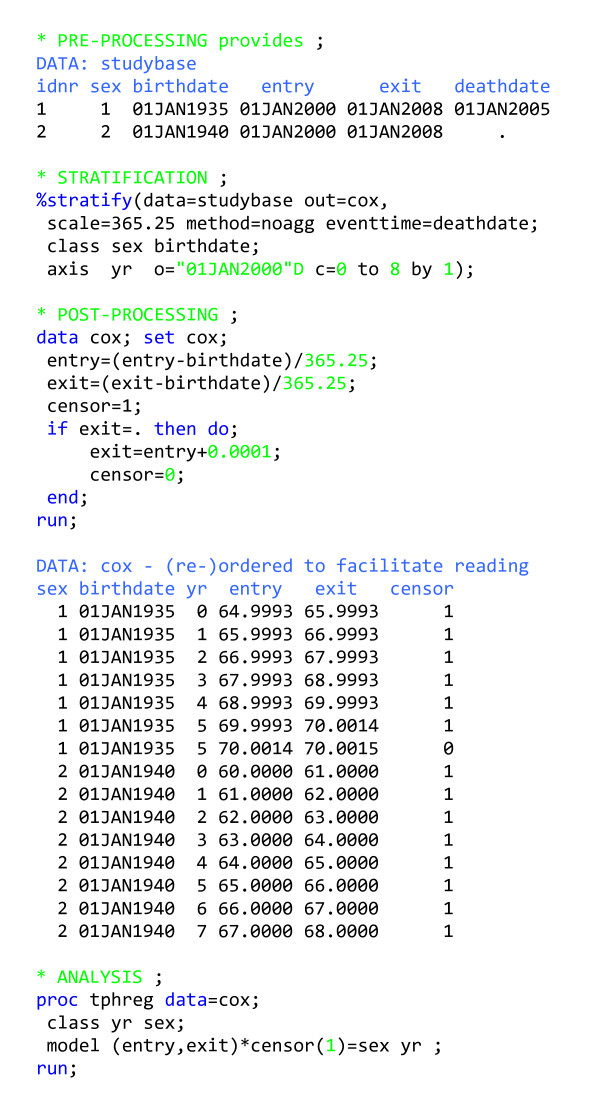
Program outline for analysing follow-up data with one outcome using Cox regression.

In figure 7, we present an exercise very similar to the one in figure 2, but this time prepare output for a Cox regression analysis with a single outcome. In some of the most popular statistical packages that allow for Cox regression with time-varying covariates (e.g. SAS[5], STATA[14] and S-PLUS[15]), follow-up time must be stratified according to any time-varying covariates ahead of time, which renders %stratify very useful. Post-processing in this case consists of calculating *entry *and *exit *on the time scale selected for the Cox analysis (age, in figure 7) and reformulating the outcome events (signalled by a missing value of *exit*) as very short follow-up intervals not ended by censoring. See [8] for examples of how to prepare output for Cox regression in modes other than (MODE = single).

### Timing problems

A common characteristic of the stratification methods presented here is that they are exact, meaning that every event and person-time contribution will be placed in the correct stratum. Many older methods for stratification are approximate, for example, only taking into account the status of an individual in mid-year (see [16] for an example). We do not think the resulting errors in covariates can be justified, given presently available computing resources.

However, using exact stratification methods requires that we know exactly when every exposure event and outcome event occurred. If we do not, we must guess or use a convention. For instance, if we only know that an event occurred in a particular calendar year but not when during the year (interval censoring), the event is usually considered to have occurred mid-year with adjustments to accommodate such constraints as having the event occur to a living person, because the exposure or outcome will otherwise not be taken into account in the analysis. The analyst should always consider in each individual study how best to handle inaccurate or erroneous timing of events.

In competing risks analyses, it is assumed that concurrent outcomes will not occur [17]. However, in practice, this occurs often. In such cases, we have to choose which of the co-occurring outcomes occurred first to ensure a simple competing risks analysis. When the macro is used with MODE = c and an external outcome event data set is specified, as in figure 4, the macro automatically picks the first outcome event for a given SUBJECT, and when two or more outcome events appear simultaneously as the first in a SUBJECT, it picks the one with the lowest EVENTTYPE. This decision rule is quick, easy and reproducible. However, depending on the study, more elaborate decision rules may have to be devised and enacted by generating an outcome data set accordingly. Coding of death certificates often involves the same kind of choices, with standardised guidelines. As an alternative to choosing one of the co-occurring outcomes over the others, some have suggested that combinations of co-occurring outcomes could be outcomes in their own right [17]. Alternatively, instead of editing the data to fit the model, the analyst could decide to perform a more complicated analysis, using all available information (see [18] for an example). In any case, it is always a good idea to start by examining the data for co-occurring outcomes.

Co-occurrence of exposure events may also cause problems. For example, if a person changes residence more than once within the finest division of time used, the analyst must ensure that only the record representing the final residence is included in the relevant exposure data set. Otherwise, the resulting person-time table may not be entirely correct.

Slight discrepancies between intended and actual cut points can occur, due to variation in calendar year length. This is especially a problem for stratifying tools that allow scaling of one set of time points (cut points) relative to another set of time points (entry, exit, origins). These effects are usually negligible, although an event occasionally appears in or disappears from a stratum due to such inaccuracies. We have no generally preferred solution for this problem. However, this general problem has important consequences for this macro, because if the entry time is missing or earlier than the earliest cut point on some time axis, a fatal error occurs. This type of error usually results from inadequate data cleaning; a data (or, alternatively, programming) error that allows follow-up to start before birth, for example, is a typical cause of a macro error of this sort. However, fatal macro errors are also commonly due to inaccuracies with regard to the calendar period time axes. One way to avoid this is to choose the origin for the time scale as the smallest cut point. For example, if we want to stratify by calendar years 1968 to 2000 we could write origin = "01JAN1968"D cuts = 0 to 32 by 1, and later recode the variable during post-processing if we wanted it to have a more familiar look. Another way to avoid this error is to have all time scales (entry, exit, origins, cut points) in decimal years. Macros for this are available at [8].

## Considerations in the choice of survival analysis model

The level of complexity of the exposure history almost completely determines the amount of work involved in a survival analysis, regardless of the tool used for analysis. Much of this tutorial is applicable to any type of survival analysis with time-varying covariates. Using Cox regression and Poisson regression to solve the same problem involve essentially the same amount of programming with our macro (compare figures 2 and 7).

In practical terms, one should not expect noticeable differences between results from similar Poisson and Cox models, as Cox regression provides fairly efficient rate ratio estimates, despite being based only on a partial likelihood [19,20]. During processing the size of the data set needed for Cox regression is proportional to the union of risk sets, while the size of the data set needed for Poisson regression is proportional to the number of non-empty combinations of levels of the covariates. Therefore, performing Cox regression can be slow compared to Poisson regression for large data sets, and the former cannot be performed at all on gigantic data sets such as the one used by Edgren et al. [21], for which Poisson regression takes a few minutes. Anything that can be done using Cox regression can also be done using Poisson regression, and the exact same results can be obtained in the absence of ties [22]. It is now possible to perform robust estimation of parameter variance [23-26] in both SAS proc genmod [27] and SAS proc phreg/tphreg [28]. In our experience, Poisson regression analysis of large, thinly-stratified event-time tables typically exhibits underdispersion as measured by the deviance but good agreement between the hypothetical (Poisson) and actual (robustly estimated) parameter estimate variances. In our experience, overdispersion and the subsequent risk of generating false positive findings, are much more common in small data sets, but in such situations, robust variance estimation is now easily accomplished.

Cox regression forces one time scale to be a response variable of special importance compared with others, and the extremely detailed modelling of this time scale can lead to over-interpretation of small details in the survival curve. Interactions with this time scale are somewhat obscure and non-parsimonious. On the other hand, Poisson regression treats all time scales equally as covariates and interactions between time scales and other covariates (and time-scales) are modelled in a simple and natural way [4]. In contrast to Cox regression, Poisson regression requires categorisation of continuous predictor variables, which creates concerns about residual confounding and other biases arising due to this loss of information. However, with modern computing hardware, it should be possible to work with such finely grouped predictor variables that these concerns become merely theoretical. For a detailed comparison of Cox regression and Poisson regression, see Carstensen [4]. Note that he does not consider aggregating data for Poisson regression, which we have shown to be very useful for large data sets.

Other analytic approaches to survival analysis often require data sets similar to those used for either Cox or Poisson regression. The type of input data set used for Markov chain Monte Carlo (MCMC) techniques and other computer-intensive methods depends on the choice of basic model. For computational reasons, it is typically advantageous to use a Cox-style input data set when analysing data from small clinical trials, whereas it is advantageous to use event-time tables when following large cohorts.

It is also usually possible to analyse follow-up data using nested case-control methods. The loss in efficiency (statistical precision) compared to using the full data set in a cohort study is usually small, and if the cohort study by design has certain virtues in terms of lack of bias etc., these properties carry over to a properly conducted nested case-control study. The data set required for analysis is often far more manageable, and assuming density sampling, the determination of the values of time-varying covariates at the time of entry into the risk set is easier than keeping track of them throughout the follow-up period. That being said, in our view, follow-up data should not generally be analysed using nested case-control methods, mainly because doing so excludes many possible modes of inquiry, such as fitting additive rate models or assessing absolute measures of disease frequency, that may yield important insights.

## Conclusion

This tutorial has explored the capabilities of a new macro designed to perform the most well-defined and standardised tasks in event-time table creation. We have demonstrated some of the new programming possibilities enabled by the macro's data interface and user interface and shown that generating event-time tables for many outcomes simultaneously (as opposed to for a single outcome) requires only a few extra lines of code. The main gain from these new programming possibilities is in efficiency, i.e. fast execution. However, that the macro avoids long lists of outcomes to be analysed, or worse still, many repetitions of almost identical code, is also of considerable importance; instead, our methodology will by default go through all the outcomes available in the relevant data files, so that particular outcomes can be dropped or manipulated in the program, but ordinary outcomes will not call attention to themselves in the program. Another novelty is that little or no ad-hoc programming is needed to handle bit-rate time factors (time factors growing alternately at the speed of time or not at all such as cumulative employment). The macro also provides flexibility in choosing cut points within and between multiple time axes, an improvement compared to programs such as the classical SAS macro by Macaluso [10], which require that all time scales have equidistant cut points with the same interval length for all time scales.

Our macro can handle time-varying zero-rate time factors, something not possible in the classic SAS macros [10,29]. In the common and simple situation of having only one type of exposure event with few attributes to consider, it may be perfectly satisfactory to stratify data in pre-processing according to zero-rate time factors. However, as soon as there are many exposure event attributes and/or several types of exposure event to consider, the advantages of the macro's capabilities – in terms of both reading and writing the code – become obvious.

## Competing interests

The author wrote the macros presented and may therefore be biased in their favour.

## Appendix

In this appendix, we compare different methods (three of our own macros and some classics) for stratifying unity-rate time factors and aggregating person-time, with respect to time and memory requirements. Our own macros are ancestors of the current stratify macro, in which the methodology for each is implemented by specifying different values of MODE. The items mentioned under "Future developments" are still on the wish list for the stratify macro.

### Algorithms

We use Macaluso's method B [10] as our algorithm for stratifying individual person-time, realising the full potential of this algorithm, which was originally described by Clayton in [3].

We have written three macros that differ only with regards to the way in which person-time is aggregated over individuals. In the simplest macro (%sumpyrs), we write each contribution to a data set and then aggregate all contributions. An alternative (%arrpyrs) is for each non-empty combination of the levels of the already stratified variables to aggregate contributions for each combination of levels of unity-rate time factors in a multidimensional array (the PY array), with dimensions governed by the product of cut points, write the contents of non-empty array cells to a data set, and re-initialise these cells. This technique requires that the input data set be sorted by all the already stratified variables and that the data be handled in chunks containing all the observations with the same values of these variables. This is called by-processing and is immediately available in SAS and is essential in order to keep the size of the PY array manageable. In data sets with many zero-rate time factors and many detailed time axes, one runs the risk of examining a large sparse PY array very frequently. To combat this problem, we wrote an extension (%fstpyrs) to %arrpyrs where we keep track of all cells visited for the present combination of levels of already stratified variables in a second multidimensional array (the pointer array), and only read and re-initialise the relevant cells when it comes time to write to the output data set.

The algorithms for aggregating person-time and events implemented in %fstpyrs and %arrpyrs improve on ideas presented by Wood *et al *[9]. Using by-processing, we can create very detailed data sets without exhausting working memory during event-time table construction.

### Performance and limitations

The merits of the three macros presented above and two widely used alternative macros were assessed on simulated data sets. The two external macros were the classic SAS realisation of the Macaluso B method described in [10] and the lexis macro [29]. The latter is similar to the stsplit function in STATA and lexis.R in R [14,30,31]. The Macaluso B macro requires that the cut points on each time axis be equidistant. The lexis macro offers full flexibility in choosing cut points, but can only stratify data one time axis at a time. Both methods require an aggregating step, realised with SAS proc summary as in the sumpyrs macro [28].

Although not strictly necessary, %sumpyrs, %lexis and Macaluso B almost always gain from using by-processing (to avoid excessive disk I/O), so we employed by-processing throughout. Thus, observed differences in total run time are due only to differences in macro stratification efficiency and to random variation. The sumpyrs macro was consistently faster than Macaluso B, illustrating the efficiency of our program for stratification of follow-up time (common to %sumpyrs, %fstpyrs, %arrpyrs). Stratifying person-time using %lexis typically took 2 to 3 times longer than the same procedure using %sumpyrs, as the lexis macro's reading and writing of ever larger data sets consumed a great deal of time.

The fstpyrs macro was consistently faster (2 to 3 times faster) than %sumpyrs. In the simplest possible situations, with very small PY-arrays, %arrpyrs was slightly faster than %fstpyrs, but in such situations, time consumption was modest anyway. When the average PY array became sparse and was evaluated frequently, time consumption for %arrpyrs became excessive (up to 100 times slower than %fstpyrs in our simulations).

To get an idea of the potential gains to be had from further improvements in stratification efficiency, we disabled output from the stratifying data step in %sumpyrs and compared the run time for the stratifying data step with the total run time for %fstpyrs. Between 14% and 38% of the run time was spent on stratification, with run times proportionally longest during the simplest scenarios; thus, when the fraction of computer run time spent on stratification is substantial, overall run time is likely to be short (i.e. the entire task is performed quickly). Consequently, algorithms requiring e.g. common interval lengths for all time axes in order to enable faster stratification [10,32] seem, therefore, to be of little practical value given current computer processing speeds.

### Future developments

In the current implementation of the fstpyrs macro, the pointer array takes up more space than the PY array whenever there are more than two time axes, to permit inclusion of every possible combination of the levels of stratified unity-rate time factors [8]. However, usually only a tiny fraction of this space is needed. It requires only a few lines of code to modify the macro to cap the size of the pointer-array with respect to the number of observations. Sensible choices for this cap should guarantee that the pointer-array is small while rarely if ever resulting in a situation in which the output data set is not as small as possible, what statisticians call minimally sufficient.

In extraordinary situations when we have to use %sumpyrs for lack of sufficient working memory (RAM) and must work on a large input data set, a prohibitively large intermediate data set (the stratified data set before aggregation) can result. To avoid this, we have created a variant of the sumpyrs macro which includes some primitive automatic logic to chop up the sorted input data set into smaller chunks for complete processing (stratification and aggregation), the results of which are then concatenated to form the output.

These two examples emphasise the desirability of a general fast algorithm for aggregation, the memory needs of which do not grow in proportion to the number of cut points per axis or the number of observations. The most promising methods for achieving this involve implementation of nearly balanced binary trees [33] (Jacob Simonsen – personal communication). However, such methods may sacrifice the guaranteed minimally sufficient event-time table in order to stay within working memory during event-time table construction, and may also require the use of facilities external to SAS.

The list of future refinements we may want to undertake is relatively short:

1) Create the necessary programming logic to make automated intelligent choices between the various algorithms for aggregation based on programming environment characteristics and the data files being processed.

2) Minimise potential side effects such as the possibility of overwriting variables intended to be in the output data set.

3) Force the macros to recognise different time scales with a common origin and combine them into a single scale during calculations.

Macro implementation has been aided by features available in the SAS language such as by-processing, multidimensional arrays and a rich macro language. The use of the SAS programming language ensures that these and other macros are immediately available to many potential users on almost any computing platform. On the other hand, the algorithms may not be that easy to implement in other languages. Nevertheless, it should be possible to use large parts of the methodology presented here even without the specific macros. We would be interested to hear from people interested in and capable of adapting parts of our macros for other languages, as we do not plan to do this ourselves.
